# A Novel Bioelectronic Reporter System in Living Cells Tested with a Synthetic Biological Comparator

**DOI:** 10.1038/s41598-019-43771-w

**Published:** 2019-05-13

**Authors:** Ji Zeng, Areen Banerjee, Jaewook Kim, Yijie Deng, Tim W. Chapman, Ramez Daniel, Rahul Sarpeshkar

**Affiliations:** 1 Departments of Engineering, Microbiology & Immunology, Physics, and Molecular and Systems Biology, Dartmouth, Hanover, New Hampshire 03755 USA; 2 Thayer School of Engineering, Dartmouth, Hanover, New Hampshire 03755 USA; 3 Molecular and Cell Biology, Dartmouth, Hanover, New Hampshire 03755 USA; 4 Technion – Israel Institute of Technology, Haifa, 3200003 Israel

**Keywords:** Applied microbiology, Synthetic biology

## Abstract

As the fields of biotechnology and synthetic biology expand, cheap and sensitive tools are needed to measure increasingly complicated genetic circuits. In order to bypass some drawbacks of optical fluorescent reporting systems, we have designed and created a co-culture microbial fuel cell (MFC) system for electronic reporting. This system leverages the syntrophic growth of *Escheriachia*. *coli* (*E*. *coli*) and an electrogenic bacterium *Shewanella oneidensis* MR-1 (*S*. *oneidensis*). The fermentative products of *E*. *coli* provide a carbon and electron source for *S*. *oneidensis* MR-1, which then reports on such activity electrically at the anode of the MFC. To further test the capability of electrical reporting of complicated synthetic circuits, a novel synthetic biological comparator was designed and tested with both fluorescent and electrical reporting systems. The results suggest that the electrical reporting system is a good alternative to commonly used optical fluorescent reporter systems since it is a non-toxic reporting system with a much wider dynamic range.

## Introduction

Biosensors play a vital role in biotechnology, including but not limited to environmental monitoring, medical diagnostics, drug discovery, process control and food safety^1^. In the last three decades, the use of microbial biosensors (whole-cell biosensors) has gained in popularity. This popularity has been largely due to their negligible adverse impact on society and the ecosystem^2–4^. Such biosensors typically rely on either biochemical reactions (colorimetry) or fluorescence for reporting^5–7^. Because these methods are relatively expensive and currently need bulky equipment like fluorescence microscopes and flow cytometry (FC), they are not well suited for many applications. They only allow for acute measurements over the course of a few hours and can lead to non-ideal conditions like photo-bleaching and toxicity, which are caused by the catalytic oxidative stress from fluorescent proteins^8^ during chronic measurements^9–11^. Cross-contamination and equipment malfunction are also common. The interpretation of absorption and emission spectra from multiple sources, and of forward-scatter and back-scatter optical measurements is also not always straightforward. Thus, there is often debate about what is being measured.

The concept of using electrons as a reporter for biochemical reactions has gained importance in the field of biosensors after the identification of certain bacterial species like *Geobacter spp*. and *Shewanella spp*. capable of extracellular electron transfer^12–15^. *Shewanella oneidensis* MR-1 is a gram-negative facultative anaerobe belonging to the γ-group of the proteobacteria. It can use more than 14 different electron acceptors during aerobic and anaerobic respiration^16–21^. This ability to transfer electrons to an electrode in the anodic side of a microbial fuel cell makes it an attractive choice for biosensors^22,23^. The transfer can occur using various mechanisms such as direct electron transfer (DET) via membrane-bound cytochromes^24,25^ and metabolite (flavin) mediated electron transfer^26–29^.

Synthetic microbial consortia have been extensively studied in metabolic engineering and synthetic biology^30–32^. Co-culture systems provide advantages over single-culture systems including the ability to tolerate higher metabolic stress, optimize biochemical reactions in different environments, and avoid intermediate toxicity^33^. Here, we use a microbial fuel cell (MFC) based on an *E*. *coli* MG1655 strain that is paired with an *S*. *oneidensis* MR-1 strain in a co-culture. We utilize this co-culture system for electrical reporting of circuits in *E*. *coli*. In this co-culture system, lactate produced by fermentation in *E*. *coli* acts as the electron donor for *S*. *oneidensis* MR-1 resulting in current production. In essence, we are measuring the current produced by *S*. *oneidensis* MR-1 as a function of a molecule generated in *E*. *coli*. As a proof-of-concept demonstration, we designed a novel synthetic biological comparator and tested this circuit with both an optical reporting system and our electrical reporting system. In the optical reporting system, the input is amplified via the transcription and translation of the comparator circuit and reported as the expression level of the fluorescent protein. In the electrical reporting system, the input is amplified via the same biological processes and reported as the expression level of LacZα. However, the enzymatic activity of LacZα provides a second level of amplification wherein one molecule of LacZα helps *E*. *coli* produce many molecules of lactate. The produced lactate is metabolized by *S*. *oneidensis* and reported as an electrical current. Therefore, the electrical reporting system acts as a two-gain amplifier^34–37^ that can provide superior dynamic range compared with an optical reporting system where the reporting molecule is only passively sensed by light without further amplification.

## Results

**Figure 1:**
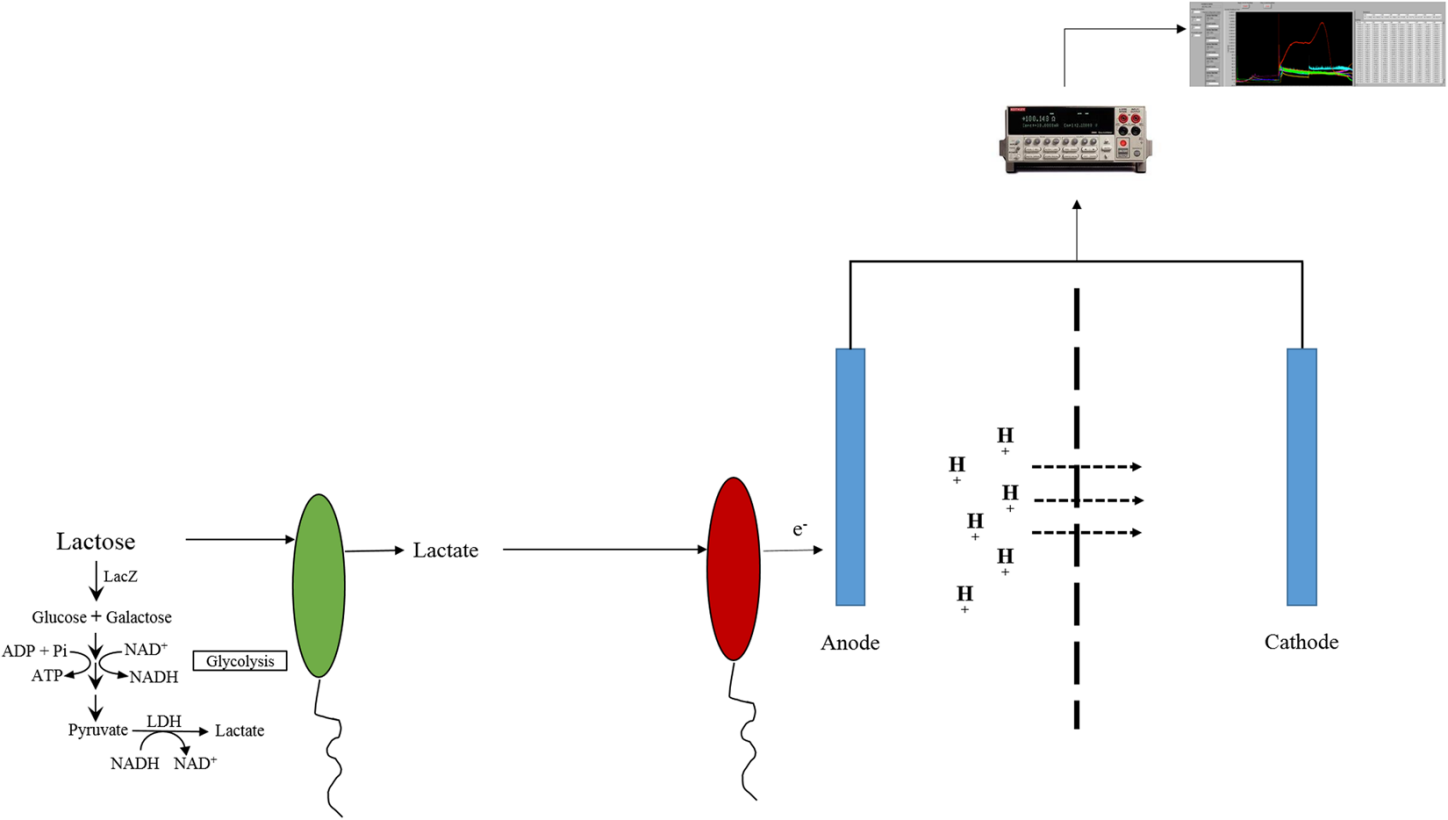
Diagram of the co-culture microbial fuel cell (MFC). The *E*. *coli* MG1655 and *S*. *oneidensis* MR-1 co-culture are grown in the anode chamber of the MFC. The green oval represents *E*. *coli* cells, while the red oval represents *S*. *oneidensis* cells. Lactose is hydrolyzed by LacZ to make glucose and galactose, which are then fermented to produce lactate in *E*. *coli*; the lactate is then utilized by *S*. *oneidensis* as its sole carbon and energy source. LDH: Lactate dehydrogenase. As *S*. *oneidensis* metabolizes lactate and donates electrons to the anode, protons cross the Nafion membrane from the anode chamber to the cathode chamber, thus creating a continuous current in the circuit. The current generated by the MFC was measured by a Keithley electrometer. Note: The picture of the electrometer was taken by Areen Banerjee and incorporated into the figure.

The anaerobic metabolism of *S*. *oneidensis* MR-1 (*S*. *oneidensis*) has been extensively studied^38–40^. Lactate, but not lactose, can be oxidized by *S*. *oneidensis* as the sole carbon and electron source under anaerobic conditions^38–40^. In addition, *S*. *oneidensis* forms syntrophic relationships with fermentative microbes under anoxic conditions^38^. Inspired by the living status of *S*. *oneidensis* in nature, an anaerobic co-culture system has been designed, in which *E*. *coli* MG1655 (*E*. *coli*) and *S*. *oneidensis* mutually benefit each other. In the anaerobic co-culture system, lactose is provided as the sole carbon source to support the growth of bacteria (Fig. 1). During fermentation, lactose is hydrolyzed by *E*. *coli* via β-galactosidase to produce D-galactose and D-glucose. These are further oxidized via the Leloir pathway and EMP pathway^41^ to produce lactate. Lactate is then secreted into the growth medium, providing a carbon source and electron source for *S*. *oneidensis*. A conductive anode as the electron acceptor for *S*. *oneidesis* and the produced current is then detected by a Keithley electrometer. Since a high lactate concentration is toxic to *E*. *coli* growth, the consumption of lactate by *S*. *oneidensis* is also beneficial to *E*. *coli*.

**Figure 2:**
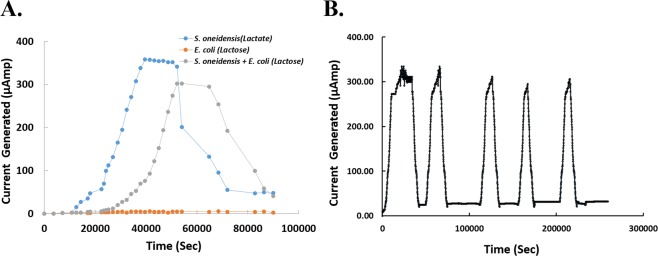
(**A**) Current generated by the MFC. Current produced by the MFC at the anode was measured in the presence of an *E*. *coli* and *S*. *oneidensis* co-culture with lactose (gray curve), an *E*. *coli* culture alone with lactose (orange curve), and an *S*. *oneidensis* culture alone with supplemented lactate (blue curve). Electrical signal is only produced in the presence of *S*. *oneidensis* (current producer) and lactate (energy and carbon source for *S*. *oneidensis*). (**B**) Test of current reproduction. Lactose was added several times, following the decay in the electrical signal, in order to demonstrate that the limiting factor of current generation is a decrease in carbon source, and not a decrease in other nutrients found in the growth medium. The peak electrical current is relatively unchanged in each cycle of lactose re-addition; thus, a cell-mass change, if any, does not affect the generation of electricity significantly in this experiment.

Figure 2A reveals electrical measurements from such a co-culture: An electrical current spike starts about seven hours after growth and disappears after about twenty-five hours (Fig. 2A, gray curve). To confirm that this spike is produced by the mutualistic growth, *S*. *oneidensis* and *E*. *coli* were cultured separately with lactose and lactate as sole carbon sources, respectively (Fig. 2A, blue and orange curves). As expected, the culture of *E*. *coli* with supplemented lactose does not produce electricity, while *S*. *oneidensis* plus lactate generates a similar spike to the consortia. Interestingly, the electricity generation of *S*. *oneidensis* is only delayed about three hours, which is half the delay time of the consortia. Such a delay may be due to the time needed for lactose metabolization and diffusion from *E*. *coli* to *S*. *oneidensis*. We also investigated the limiting factor that caused the observed drop in current in Fig. 2A. The addition of lactose was repeated after the complete disappearance of current (Fig. 2B); the MFC was able to re-generate the current spike indicating that lactose was indeed the limiting factor.

As the field of synthetic biology continues to grow, new measurement tools with low toxicity, wide dynamic range, and good sensitivity are needed^42^. Our system of electrical reporting alleviates the toxicity caused by optical fluorescence. In addition, any promoter or genetic circuit using *lacZα* as the output can be measured by this system, as the concentration of LacZα is proportional to the electric current generated by *S*. *oneidensis*. In our system, relatively low gene expression is amplified by the enzymatic activity of LacZα.

**Figure 3:**
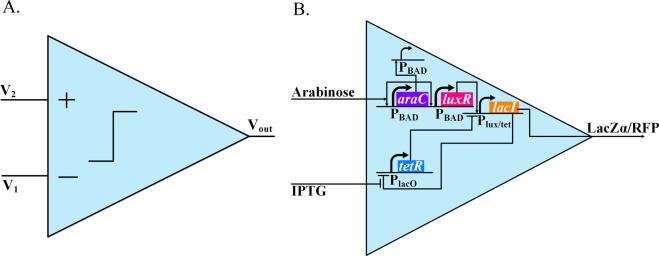
(**A**) Electronic Comparator. A comparator compares the intensity of two input signals, producing a digital output that is on when V_2_ is larger than V_1_ and off when V_2_ is smaller than V_1_. (**B**) Biological Comparator. The comparator compares the intensity of two input inducer signals (Arabinose & IPTG), turning on when the arabinose concentration is sufficiently higher than the IPTG concentration, and off when the arabinose concentration is sufficiently lower than the IPTG concentration.

**Figure 4:**
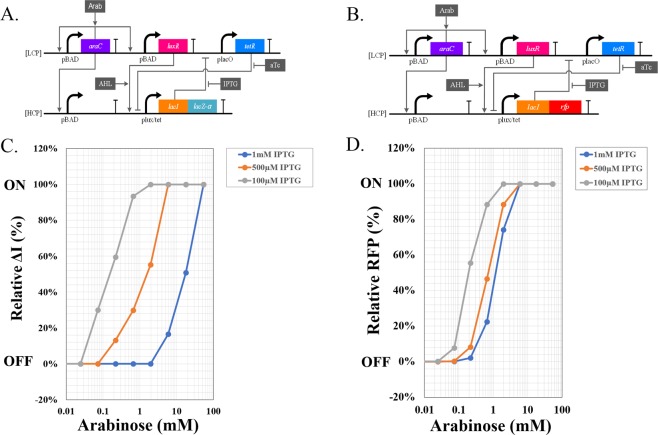
(**A**,**B**) The synthetic biological circuits used in the biological comparator with an electrical reporter system (**A**) and an optical fluorescent reporter system (**B**). LuxR is activated by a log linear AraC circuit^43^. LacI is repressed by TetR, and activated by LuxR via a hybrid promoter P_lux/tet_. Arabinose (Arab) and IPTG compete with each other to switch *lacI* on or off respectively. The on/off status of *lacI* is electrically reported via *lacZα* (**A**) or optically reported via *rfp* protein (**B**). LCP refers to ‘Low Copy Plasmid’ JF77; HCP refers to ‘High Copy Plasmid’ JF216 (**A**) or JF72 (**B**). (**C**,**D**) The behavior of the Biological Comparator when measured with the electrical reporter system (**C**) or an optical fluorescent reporter system (**D**). For the electrical reporting comparator, arabinose was added with different concentrations, and the current generated by MFC was recorded as the ΔI (current peak value – current base value). The “on” status was defined as ΔI equal or larger than 0.8 μAmp (recorded as 100%), while the “off” status was defined as ΔI equal or smaller than 0.1 μAmp (recorded as 0%). For the electrical reporting comparator, arabinose was added with different concentrations, and the fluorescence was measured by Molecular SpectraMax Paradigm Multi-Mode Microplate Reader.

To test our system, a biological comparator was designed and tested using both a traditional fluorescence system and our electrical reporting system. In electronics, a comparator is a device that compares the voltage of two input terminals, and outputs a digital on/off signal (Fig. 3A). In our biological setting, a comparator is a genetic circuit that compares two biochemical molecules, and outputs a digital on/off signal via a reporter gene (Fig. 3B). In the biological comparator, AraC, controlled by a positive-feedback-loop-and-shunt system^43^, activates wide-dynamic range log-linear analog *luxR* expression^43^. TetR is repressed by LacI, while LacI is repressed by TetR and activated by LuxR via a hybrid promoter P_lux/tet_; thus, the the *lacI* expression is digital^44^. The on/off status of *lacI* expression is determined by the competition between TetR and LuxR. Because the repression of TetR to LacI is regulated by IPTG, and the activation of LuxR to LacI is controlled by arabinose, the concentration of these two inputs (IPTG/arabinose) subsequently decide the on/off status of LacI. If the arabinose/IPTG concentration ratio is sufficiently high, the *lacI* is on; otherwise, it remains off. In order to report the on/off status, either a *lacZα* gene (Fig. 4A), an electric reporter, or an *rfp* gene (Fig. 4B) was co-expressed with *lacI*. The two reporter systems were tested using a Microplate reader and our MFC reporter system.

Figure 4C shows the behavior of the comparator under differing concentrations of IPTG and arabinose, using the MFC detection system. Similarly, Fig. 4D shows the behavior of the circuit using optical detection. As shown in Fig. 4, the on/off status of *lacI* is decided by both IPTG and arabinose. At low IPTG concentration, the repression of TetR to LacI is weakened due to the mutual repression mechanism, therefore, relatively low arabinose is able to turn on *lacI*, and subsequently the co-expressed *lacZα* (Fig. 4C) and *rfp* (Fig. 4D). However, the threshold shifts with the increase of IPTG concentration, which causes a higher arabinose concentration that is required to turn on *lacI*. While the fluorescent reporter system shows similar comparator behavior, the dynamic range is not comparable to electric reporter system. This is because the co-culture MFC system has greater output amplification. The output of the electric reporter system is an enzyme rather than a fluorescent protein. This increases the gain as a single LacZα molecule can produce thousands of lactate molecules and subsequently electrons and electrical current.

## Discussion

Fluorescent reporting systems are the most widely used reporting systems in biological experiments due to their convenience for design and application, stability, and their ability to be quantified. However, as synthetic biology produces increasingly complex circuit topologies for in-the-filed applications, unobtrusive detectors capable of chronic reporting and ultra-low signal detection are needed^42,45,46^. In order to achieve these goals, we have designed and constructed a co-culture microbial fuel cell and converted it into an electric reporting system. Compared to a fluorescence-based system, the co-culture electric reporter does not need to expose cells to the toxicity of optical fluorescence and is therefore suitable for chronic measurement of circuit performance^42,45,46^. In addition, this system gives a wider dynamic range compared to optical systems (Fig. 4C,D). As we have demonstrated, the 1 mM IPTG curve is not discernible from the 500 μM IPTG curve by fluorescence (Fig. 4D) but is easily visible using the electrical reporting system (Fig. 4C). As any electronic/biological circuit, there is a trade-off among power consumption, speed, and precision^34–37,47^. Our electrical reporting system is an example wherein higher precision is achieved at the expense of speed (Fig. 2) compared with the optical reporting system. Depending on the application, both systems can be useful and fulfill complementary needs.

The ability of a comparator to convert continuous analog signal into a discrete digital output makes it one of the most useful electronic devices^34–37^. Similarly, our biological comparator converts continuous biochemical inducer concentrations into discrete digital electrical/optical on/off outputs, which can have important medical and biotechnological applications^1^. In our biological comparator, the threshold can be finely tuned by adjusting inducer concentrations (Fig. 4). As described, TetR and LacI are mutually repressing each other. As the concentration of IPTG increases, the ability of TetR to repress LacI is strengthened, changing the flip point of the *lacI* on/off expression. In addition, this comparator circuit is very robust to the variations of AHL and ATc concentrations as we demonstrate in Supplementary Fig. 1.

## Materials and Methods

### Microbial fuel cell (MFC)

*Shewanella oneidensis* MR-1 and *Escherichia coli* Strain K12 MG1655 (*F*- *lambda*- *ilvG*- *rfb*-*50 rph*-*1*) were used to test MFC cell current production (Fig. 2). *S*. *oneidensis* cells were shaken at 270 rpm and 30 °C overnight in 50 mL minimal medium^48^ supplemented with 20 mM lactate and 0.02% casamino acids. The next day, overnight culture was spun down and resuspended in 5 mL minimal medium^48^ without lactate and casamino acids. *E*. *coli* cells were shaken at 270 rpm and 37 °C overnight in 5 mL minimal medium^45^ supplemented with lactose as the carbon source. The next day, *E*. *coli* cells were washed twice with 5 mL minimal medium without a carbon source, and finally re-suspended in 10 mL minimal medium without a carbon source. The OD600 of both strains were measured. The two cultures were added to a 250 ml Basal medium with lactose and 0.02% casamino acids with the ratio of *S oneidensis* to *E coli* being at 1000:1.

The latter co-culture was added to the anode side of the microbial fuel cell. The cathode contained *Potassium Ferricyanide* (K_3_FeCN_6_) solution. The electrodes for the anode and cathode were carbon cloth and the cation exchange membrane was a Nafion membrane (CMI-7000S from Membranes International Inc.). The MFC was connected to a source meter and the electricity generated by the device was tracked for a further 48–72 hrs. to confirm that all the lactose in the anode was depleted (indicated by drop in the electricity generation to 7 µAmp or less).

### DNA manipulation and plasmid construction

All enzymes for DNA manipulation were from New England Biolabs unless stated otherwise. Phusion DNA polymerase (New England Biolabs) was used for all DNA amplifications, except for colony PCR where we used Qiagen *Taq* polymerase. Plasmids, PCR products, and DNA fragments from agarose gel were purified with Qiagen miniprep, PCR purification, and Gel extraction kits, respectively.

A low copy plasmid (JF77) and a high-copy plasmid (JF216 (electrical) or JF72 (optical)), as shown in Fig. 4A,B, were constructed to implement our circuits. The primers and parts for the synthetic circuit for expression of LacZα and RFP were designed and ordered as gBlocks (IDT DNA). The plasmid backbone and parts were put together using a Gibson Assembly kit (New England Biolabs). The resulting plasmids JF72, JF77, and JF216 were sequenced and confirmed to be accurate using commercially available sequencing services (Genewiz).

### Fluorescent reporting of biological comparator circuit

*E*. *coli* strain NEB10β (*araD139* Δ(*ara*-*leu*) *7697 fhuA lacX74 galK* (ϕ*80* Δ(*lacZ*) *M15*) *mcrA galU recA1 endA1 nupG rpsL* (Str^R^) Δ(*mrr*-*hsdRMS*-*mcrBC*)) (New England Biolabs) was used for fluorescent reporting of comparator circuit (Fig. 4). *E*. *coli* cells transformed with biological comparator with RFP reporter were shaken at 250 rpm overnight, 37 °C, in LB medium with 50 μg/mL kanamycin and 50 μg/mL carbenicillin. The next day, overnight culture was inoculated 1:100 ratio in 3 mL fresh LB medium with 50 μg/mL kanamycin and 50 μg/mL carbenicillin, and shaken at 250 rpm, 37 °C, for 25 minutes. 100 μg/mL AHL, and three different IPTG concentrations (1 mM, 500 μM, and 100 μM) were added to the inoculations. The culture with appropriate AHL and IPTG concentrations were then aliquoted to 96 well plates, and 0.8% Arabinose was 1:3 serial diluted to make the final circuit growth condition. After addition with the chemical inducers, inoculations were shaken at 700 rpm, 37 °C, for 4 hours in VWR 1585 Microplate shaker. The RFP molecular concentration was then measured by Molecular SpectraMax Paradigm Multi-Mode Microplate Reader.

### Electrical reporting of biological comparator circuit

*E*. *coli* strain NEB10β (*araD139* Δ(*ara*-*leu*) *7697 fhuA lacX74 galK* (ϕ*80* Δ(*lacZ*) *M15*) *mcrA galU recA1 endA1 nupG rpsL* (Str^R^) Δ(*mrr*-*hsdRMS*-*mcrBC*)) (New England Biolabs) was used to test electrical reporting of biological comparator circuit (Fig. 4). *E*. *coli* cells containing the comparator circuit with LacZα reporter were grown according to the protocol mentioned above (Fluorescent reporting of biological comparator circuit) with minor modifications. Minimal medium supplemented with lactose as the sole source of Carbon was used for growth. The samples were centrifuged at 10000 rpm for 15 minutes and the supernatant was passed through a 0.2 µm filter. The samples were placed at 4 °C until ready to be analyzed. Before analysis the samples were brought to room temperature. 0.3 ml of the collected supernatant was injected into the lactate starved microbial fuel cell (MFC).

For the lactate starved MFC, frozen *S*. *oneidensis* cells were thawed and added to fresh 6 ml LB medium in polypropylene culture tubes (VWR). This tube was incubated overnight at 30 °C under shaker conditions (270 rpm). Next day, the media containing the bacterial cultures was pooled and centrifuged at 5000 rpm in a VWR centrifuge for 25 minutes. The supernatant was discarded, and the pellet re-suspended in 30 ml basal medium (without lactate or casamino acids). The solution was centrifuged again at 5000 rpm for 25 minutes. After discarding the supernatant and drying the pellet, it was re-suspended in 30 ml basal medium supplemented with 10 mM lactate and casamino acid. The culture was incubated at room temperature for 48 hours. After incubation, the culture was centrifuged as described previously. After the second centrifugation step, the pellet was re-suspended in 20 ml basal medium (without lactate and casamino acid) and centrifuged again for 25 minutes at 6000 rpm. The pellet from this step was re-suspended in 250 ml basal medium containing 0.1 mM lactate and 0.02% casamino acid. This culture was added to the anode side of the microbial fuel cell. The cathode contained *Potassium Ferricyanide* (K_3_FeCN_6_) solution. The electrodes for the anode and cathode were carbon cloth and the cation exchange membrane was a Nafion membrane (CMI-7000S from Membranes International Inc.). The MFC was connected to a source meter and the electricity generated by the device was tracked for a further 48–72 hours to confirm that all the lactose in the anode was depleted (indicated by drop in the electricity generation to 7 µAmp or less).

## Supplementary information

Supplementary Figure S1
