# Supplementary material for: A Novel Bioelectronic Reporter System in Living Cells Tested with a Synthetic Biological Comparator
Source: Sci Rep. 2019 May 13;9:7275. doi: 10.1038/s41598-019-43771-w (PMC6513987; doi:10.1038/s41598-019-43771-w)
Supplement: Supplementary file 1 — Supplementary Figure S1 [file 41598_2019_43771_MOESM1_ESM.pdf]

A Novel Bioelectronic Reporter System in Living Cells Tested with a Synthetic Biological Comparator

Ji Zeng[b,1]; Areen Banerjee[b,1]; Jaewook Kim[b,1]; Yijie Deng[b], Tim W. Chapman[c], Ramez Daniel[d]; Rahul Sarpeshkar [a, *]

[a] Departments of Engineering, Microbiology & Immunology, Physics, and Molecular and Systems Biology, Dartmouth, Hanover, New Hampshire, 03755, USA.

[b] Thayer School of Engineering, Dartmouth, Hanover, New Hampshire, 03755, USA.

[c] Molecular and Cell Biology, Dartmouth, Hanover, New Hampshire, 03755, USA.

[d] Technion – Israel Institute of Technology, Haifa, 3200003, Israel

[1] Equal Contribution

[*] Correspondence and requests for materials should be addressed to R. S. (rahul.sarpeshkar@dartmouth.edu)

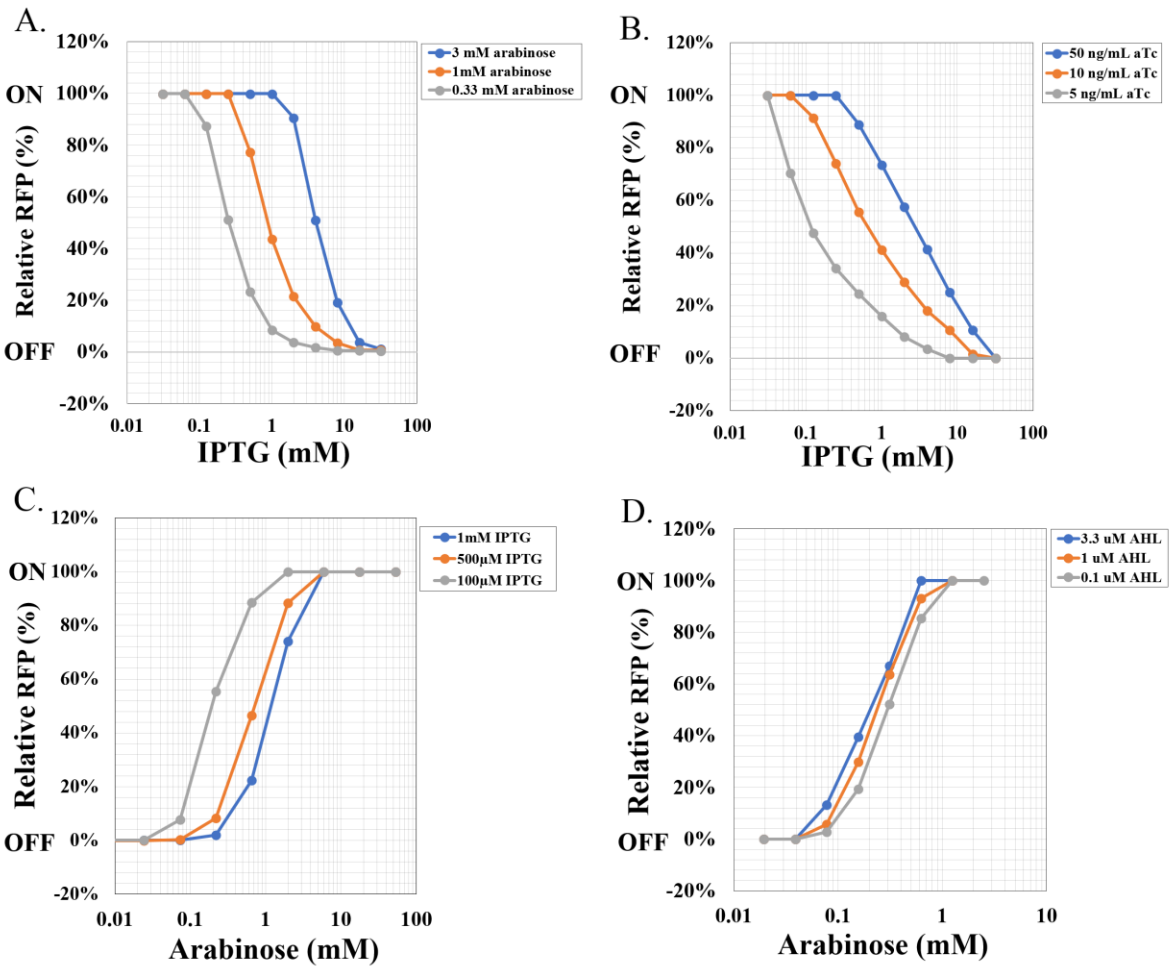

Supplementary Figure 1. Characterization of the synthetic comparator. The synthetic comparator is robust to concentration changes of IPTG, arabinose, AHL, and aTc. Increasing IPTG weakens the repression of LacI to TetR, and therefore, decreases the expression of RFP; on the contrary, increasing arabinose strengthens the repression of LacI to TetR, and increases the expression of RFP. **A and B. The synthetic comparator performance with IPTG as the switching input.** A. Increasing arabinose shifts the Kd to the right. B. Increasing aTc shifts the Kd to the right. **C and D. The synthetic comparator performance with arabinose as the switching input.** C. Increasing IPTG shifts the Kd to the right. D. Increasing AHL shifts the Kd to the left.
